# Supplementary material for: Enhanced on-chip phase measurement by inverse weak value amplification
Source: Nat Commun. 2021 Oct 29;12:6247. doi: 10.1038/s41467-021-26522-2 (PMC8556267; doi:10.1038/s41467-021-26522-2)
Supplement: Supplementary file 1 — Supplementary Information [file 41467_2021_26522_MOESM1_ESM.pdf]

Enhanced On-Chip Phase Measurement by Inverse Weak Value Amplification

Meiting Song[1], John Steinmetz[2], Yi Zhang[1], Juniyali Nauriyal[1,3], Kevin Lyons[4], Andrew N. Jordan[5,2], Jaime Cardenas[1,2,*]

[1] The Institute of Optics, University of Rochester, Rochester, NY 14627, USA.
[2] Department of Physics and Astronomy, University of Rochester, Rochester, NY 14627, USA.
[3] Department of Electrical and Computer Engineering, University of Rochester, Rochester, NY 14627, USA.
[4] Hoplite AI, 2 Fox Glen Ct., Clifton Park, NY 12065, USA.
[5] Institute for Quantum Studies, Chapman University, Orange, CA, 92866, USA.
*Email: jaime.cardenas@rochester.edu

Supplementary Note 1: Weak value amplification with free space Sagnac interferometer

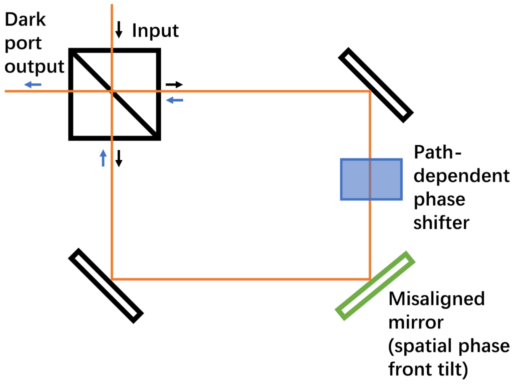

Fig 1. Free space weak value amplification with Sagnac interferometer

In previous works in free space environment, IWVA (inverse weak value amplification) was achieved with a misaligned Sagnac interferometer (Fig.1). The goal is to measure the relative phase shift ϕ between the clockwise and counterclockwise paths. The misalignment introduces a phase front tilt k to one path of the interferometer and $-k$ to the other.

$$\psi_{\pm}(x) = \phi_0(x)e^{\pm i\left(kx-\frac{\phi}{2}\right)}. \tag{1}$$

When the two paths interfere at the beam splitter, considering a Gaussian input, the dark port becomes,

$$\psi_{\mathrm{D}}(x) \propto \psi_+(x) - \psi_-(x) = \frac{e^{-\frac{x^2}{4\sigma^2}}}{(2\pi\sigma^2)^{\frac{1}{4}}}\sin\left(kx - \frac{\phi}{2}\right). \tag{2}$$

By measuring the mean location shift $-\phi/(2k)$ of the dark port spatial pattern, phase shift ϕ is determined, supposing k is known. The phase shift ϕ is amplified by $-1/(2k\sigma)$, but with the optical power reduced by $(k\sigma)^2$.

To bring free space IWVA to integrated photonics regime, the above expressions are expanded into Hermite-Gaussian (HG) modes. The beams are mainly HG_1 mode with a small contribution

of HG_0 mode. Contribution of the higher modes is negligible. Therefore, the phase front tilt can be considered as coupling the initial HG_0 mode partially into HG_1 mode.

$$\psi_{\pm}(x) \approx \left(1 \mp i\frac{\phi}{2}\right)\mathrm{HG}_0 \pm ik\sigma\mathrm{HG}_1, \tag{3}$$

$$\psi_{\mathrm{D}}(x) \propto -\frac{\phi}{2}\mathrm{HG}_0 + k\sigma\mathrm{HG}_1. \tag{4}$$

As eigenmodes of a waveguide are similar to Hermite-Gaussian modes, we applied the theory on waveguide eigenmodes TE_0 and TE_1.

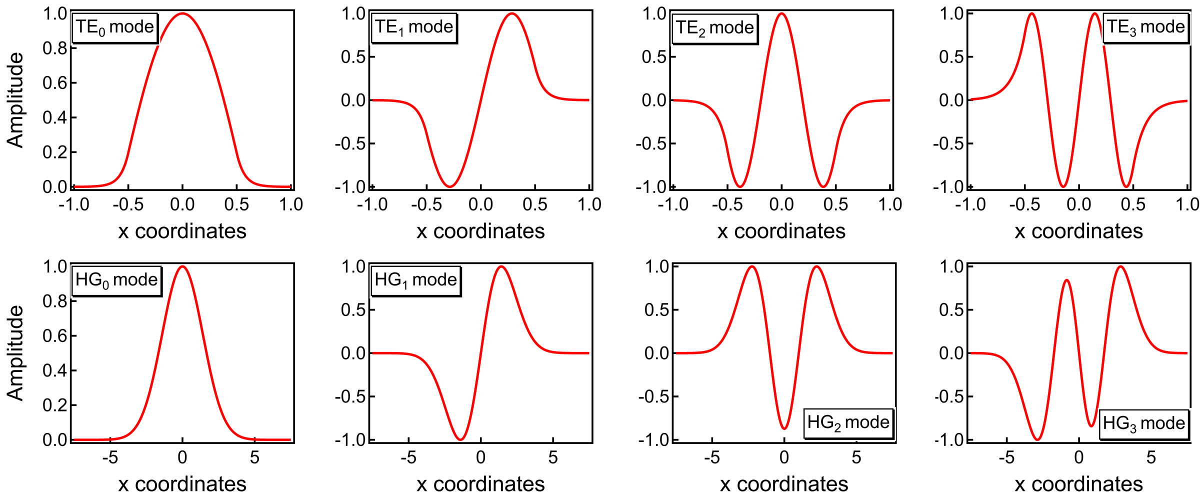

Fig 2. Electric field of TE modes in waveguides and Hermit-Gaussian modes in free space. The x coordinates are normalized. For TE modes, x is normalized to the width of the waveguide. For HG modes, x is normalized to the width of the input gaussian beam.

Supplementary Note 2: Phase front tilt calculation in a waveguide

Let us consider a one-dimensional slab waveguide structure, with silicon nitride guiding layer (2.5μm) and silicon dioxide cladding. With wavelength of λ=1550nm, the effective index of the TE_0 and TE_1 modes are $n_{\mathrm{eff},0}$ = 1.9815 and $n_{\mathrm{eff},1}$ = 1.9255. The TE_0 and TE_1 mode electric fields are

$$\mathrm{TE}_0 = \begin{cases} A_{01}e^{-\gamma_0 x}, & x \geq \frac{w}{2} \\ A_{02}\cos(\kappa_0 x), & -\frac{w}{2} \leq x \leq \frac{w}{2} \\ A_{03}e^{\gamma_0 x}, & x \leq -\frac{w}{2} \end{cases} \tag{5a}$$

$$\mathrm{TE}_1 = \begin{cases} A_{11} e^{-\gamma_1 x}, \ x \geq \frac{w}{2} \\ A_{12} \sin(\kappa_1 x), -\frac{w}{2} \leq x \leq \frac{w}{2} \\ A_{13} e^{\gamma_1 x}, \ x \leq -\frac{w}{2} \end{cases} \tag{5b}$$

$$with \begin{cases} \gamma_i = \frac{2\pi}{\lambda}\sqrt{n_{\mathrm{eff},i}^2 - n_{\mathrm{c}}^2} \\ \kappa_i = \frac{2\pi}{\lambda}\sqrt{n_{\mathrm{w}}^2 - n_{\mathrm{eff},i}^2} \end{cases}. \tag{5c}$$

z is the propagation distance. A_{ij} is normalizing constants determined by the boundary conditions.

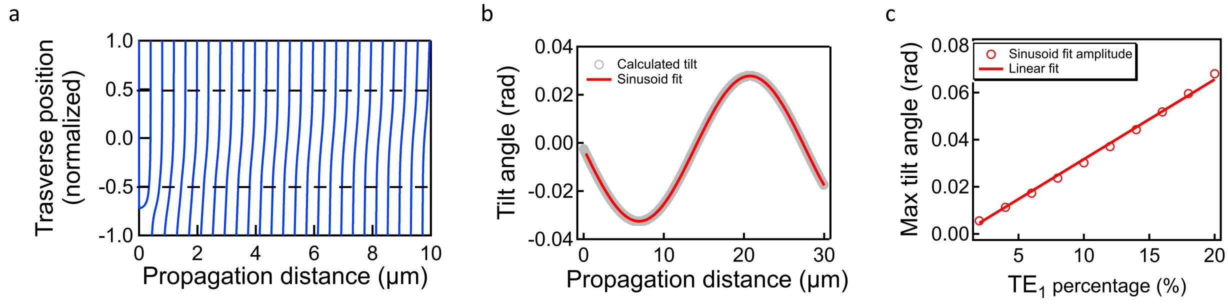

Fig 3. **a** Contour map of electric field phase along propagation ($a = 30\%$ in this figure to exaggerate the effect). The transverse coordinates are normalized to the width of the waveguide. The boundary of the waveguide is denoted by the dashed line. **b** $a = 10\%$. Tilt angle at center of the waveguide along propagation fitted to sinusoid function. **c** The maximum tilt angle (the amplitude of the tilt angle fit) for different TE_1 mode percentage.

Consider a combination of the two fields with amplitude ratio of $\mathrm{TE}_0 : \mathrm{TE}_1 = (1-a) : a$. The electric field becomes

$$E = (1-a)\mathrm{TE}_0 \cdot e^{-in_{\mathrm{eff},0}\frac{2\pi}{\lambda}z} + a\mathrm{TE}_1 \cdot e^{-in_{\mathrm{eff},1}\frac{2\pi}{\lambda}z}. \tag{6}$$

We calculate the phase of the field as it propagates in the waveguide and plot a contour map in Fig.3a. We take the center of the waveguide, where the tilt is close to linear, and calculate the tilt angle. The phase front tilt changes in a sinusoid manner with a period same as the beating of the TE_0 and TE_1 modes (Fig.3b). The maximum tilt angle (the amplitude of the tilt angle fit) increases linearly with the TE_1 mode amplitude a (Fig.3c).

Supplementary Note 3: Inverse weak value amplification with integrated Mach-Zehnder interferometer

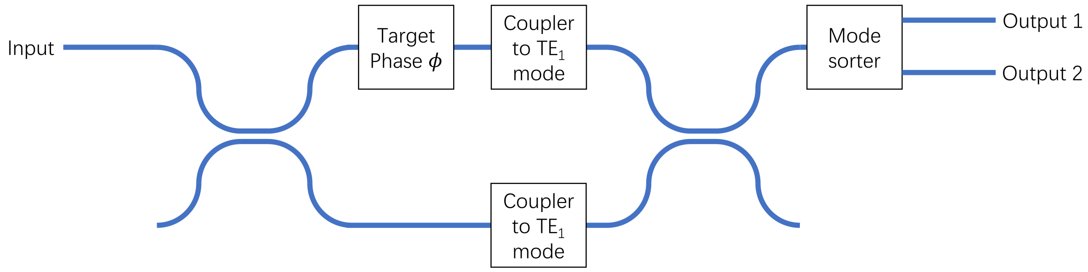

Fig 4. Schematic of the weak value device.

Assume we send a TE_0 mode into the upper waveguide of Fig.4. Since the thickness of the waveguide stays constant, we only consider the variation of field in the direction of the waveguide width, which is x axis in our notation. The input power gets split half with the appropriate length of hybridization and the fields become

$$E_1 = \frac{1}{\sqrt{2}}\mathrm{TE}_0(x), \tag{7a}$$

$$E_2 = \frac{1}{\sqrt{2}}\mathrm{TE}_0(x), \tag{7b}$$

in the two waveguides.

Then a relative phase ϕ between the two paths is added,

$$E_1 = \frac{e^{i\frac{\phi}{2}}}{\sqrt{2}}\mathrm{TE}_0(x), \tag{8a}$$

$$E_2 = \frac{e^{-i\frac{\phi}{2}}}{\sqrt{2}}\mathrm{TE}_0(x). \tag{8b}$$

Similar to free space case, part of the TE_0 mode is coupled to the TE_1 mode with opposite phase to the two paths. a is the amplitude of TE_0 mode coupled to TE_1 mode, which is taken to be a small number.

$$E_1 = \frac{e^{i\frac{\phi}{2}}}{\sqrt{2}}[(1-a)\mathrm{TE}_0(x) + ia\mathrm{TE}_1(x)], \tag{9a}$$

$$E_2 = \frac{e^{-i\frac{\phi}{2}}}{\sqrt{2}}[(1-a)\mathrm{TE}_0(x) - ia\mathrm{TE}_1(x)]. \tag{9b}$$

After the two paths interfere at the second 50/50 multi-mode splitter, the "dark port" becomes,

$$E_\mathrm{d} = \frac{E_1 - E_2}{\sqrt{2}} = i\left[(1-a)\mathrm{TE}_0(x)\sin\frac{\phi}{2} + a\mathrm{TE}_1(x)\cos\frac{\phi}{2}\right], \tag{10a}$$

$$E_{\mathrm{b}} = \frac{E_1 + E_2}{\sqrt{2}} = i\left[(1-a)\mathrm{TE}_0(x)\cos\frac{\phi}{2} - a\mathrm{TE}_1(x)\sin\frac{\phi}{2}\right]. \quad (10b)$$

Note $E_{\mathrm{b}} \approx i\mathrm{TE}_0(x) + \mathrm{O}(a\phi)$, so it contains hardly any information about ϕ.

Since ϕ is very small,

$$E_{\mathrm{d}} \approx i\left[(1-a)\frac{\phi}{2}\mathrm{TE}_0(x) + a\mathrm{TE}_1(x)\right] = ia\left[\mathrm{TE}_1(x) + \frac{1-a}{a}\frac{\phi}{2}\mathrm{TE}_0(x)\right]. \quad (11)$$

Therefore, by analyzing the ratio between TE_0 and TE_1 mode, phase ϕ can be determined. Similar to before, ϕ is amplified by $1/a$.

Supplementary Note 4: Amplification between standard MZI and weak value device

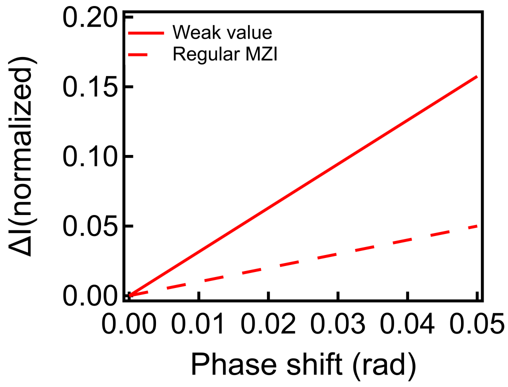

Fig 5. Optical power signal (difference of two output waveguides) of standard MZI and weak value device with respect to the phase shift. The optical power is normalized to the total optical power on the detector.

For a standard MZI interferometer in quadrature, the phase shift is determined by the output power.

$$I = I_0\left(\frac{1}{2} \pm \frac{1}{2}\sin(\phi)\right). \quad (12)$$

I_0 is total input optical power and ϕ is phase difference between the two paths. To compare with the weak value device, we also take the power difference of the two outputs as the signal.

$$\Delta I = I_0 \sin(\phi). \quad (13)$$

We take the approximation that ϕ is close to zero and normalized the power difference to the detected power, the signal becomes

$$\Delta I_{\mathrm{MZI}} = \frac{\Delta I}{I_0} = \sin(\phi) \approx \phi. \quad (14)$$

In the weak value device, we take the power difference of the two outputs of the MMI as the signal. To match the detected optical power to the MZI, the signal is normalized to the optical power in

the dark port, which is also the input optical power into the MMI. We apply a linear fit of the simulation results to the following equation

$$p = c * (I_{\mathrm{MMI1}} - I_{\mathrm{MMI2}}) = c * \Delta I_{\mathrm{wv}} \tag{15}$$

where p is the TE_0 field percentage in the input field, I_{MMI1} and I_{MMI2} are the power outputs of the two waveguides of the MMI. The difference between I_{MMI1} and I_{MMI2} is also the output of the entire weak value device, which we denote as ΔI_{wv}. The fitting parameter is $c = 1.11$.

In the simulation of the MMI outputs, we obtain its relation to the input TE_0 mode amplitude ratio ($\mathrm{TE}_0 : \mathrm{TE}_1 = p : (1-p)$). Then from Eqn. (11), we also obtain the input ratio of the two modes into the MMI.

$$\frac{\mathrm{TE}_0}{\mathrm{TE}_1} = \frac{1-a}{a}\frac{\phi}{2} = \frac{p}{1-p} \, . \tag{16}$$

Therefore, we obtain the relationship between the phase shift and the p and a parameters

$$\phi = 2\frac{p}{1-p}\frac{a}{1-a} \, . \tag{17}$$

We convert the MMI input mode parameter p (Eqn. 15) to phase shift and normalize the signal to total detected power. The optical power difference normalized to the total detected power of weak value device is

$$\Delta I_{\mathrm{wv}} = \frac{0.9}{1 + \frac{2a}{\phi(1-a)}} \, . \tag{18}$$

For the same small phase shift ϕ, the ratio of weak value device output and regular MZI output is

$$\frac{\Delta I_{\mathrm{wv}}}{\Delta I_{\mathrm{MZI}}} = \frac{0.9}{\phi + \frac{2a}{(1-a)}} \, . \tag{19}$$

Since $\phi \ll a/(1-a)$,

$$\frac{\Delta I_{\mathrm{wv}}}{\Delta I_{\mathrm{MZI}}} = \frac{0.45(1-a)}{a} \, . \tag{20}$$

Comparing the optical power signal of the standard MZI and the weak value device, the calculated amplification is 3.15 in Fig.5, which corresponds to a 9.97dB increase of the spectrum analyzer signal.

Supplementary Note 5: Micro heater and phase shift

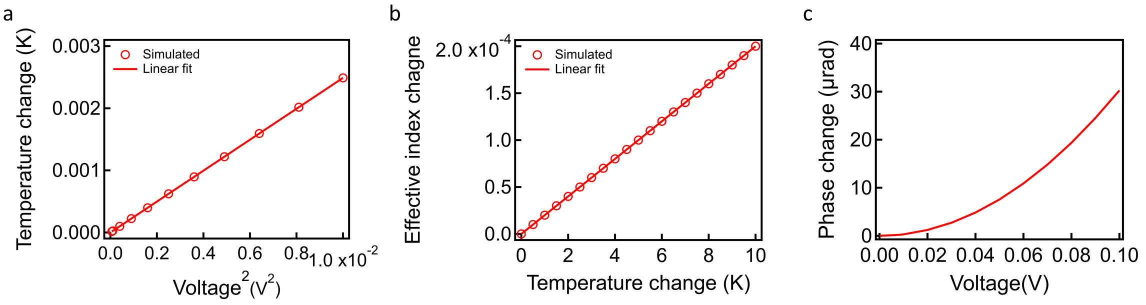

Fig 6. **a** Temperature change vs. square of applied voltage on heater. **b** Effective index change vs. temperature change. **c** Phase change vs. applied voltage on heater.

We simulate and calculate the phase change with respect to the applied voltage on the micro heater. We first simulate the heat transfer of the structure with COMSOL and get the temperature at the waveguide. Then we use the thermo-optic coefficient of silicon nitride and silicon dioxide to simulate the effective index change of the waveguide mode. The refractive indices of silicon nitride and silicon dioxide increase linearly with temperature. The thermo-optic coefficients of the waveguide materials are $2.45*10^{-5}K^{-1}$ for silicon nitride and $0.95*10^{-5}K^{-1}$ for silicon dioxide[1]. Finally, we calculate the propagation phase change with the following equation:

$$\Delta\phi = \Delta n_{\mathrm{eff}} \frac{2\pi}{\lambda} * L_{\mathrm{heater}} \tag{21}$$

where $\Delta\phi$ is the phase change, Δn_{eff} is the effective index change, λ is the wavelength, and L_{heater} is the length of the heater.

Supplementary Note 6: Frequency sensitivity vs. quality factor

In our frequency measurement setup, we use a ring resonator to convert frequency change to phase change and measure the phase change with our IWVA device. The amount of phase change introduced by a ring resonator is related to the frequency of the light and quality factor of the ring. Since we are modulating the frequency of the light, we examine the slope of the phase-frequency curve to represent the strength of the signal. As the quality factor of the ring increases, the phase introduced by the ring also increases linearly. Furthermore, the measured signal of output power difference also increases linearly with phase change. Therefore, the sensitivity of the frequency measurement should increase linearly with quality factor of the ring resonator.

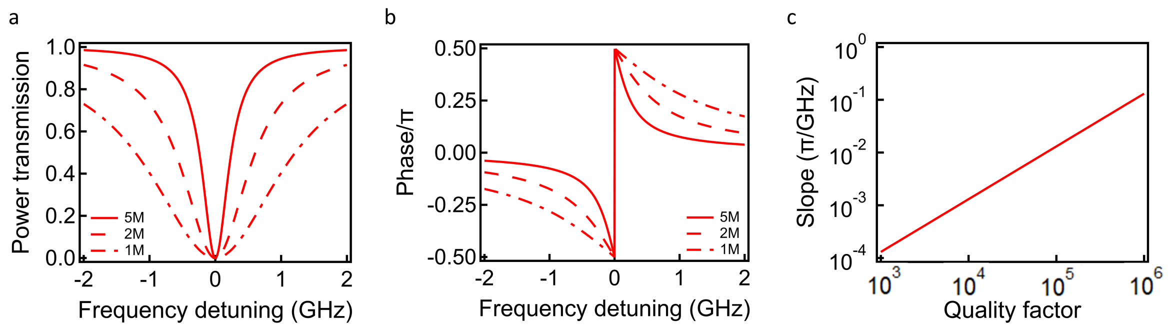

Fig 7. **a** Power transmission vs optical frequency of ring resonators with different quality factors (1, 2, and 5 million). The center frequency corresponds to the frequency of wavelength of 1550nm. **b** the phase introduced by the three ring resonators in figure a. **c** The slope of phase-frequency curve at frequencies when transmission of the ring is 0.5 plotted with respect to quality factor.

Supplementary References

1. Arbabi, A. & Goddard, L. L. Measurements of the refractive indices and thermo-optic coefficients of Si_3N_4 and SiO_x using microring resonances. *Opt. Lett., OL* **38**, 3878–3881 (2013).
